# Google Trends correlation and sensitivity for outbreaks of dengue and yellow fever in the state of São Paulo

**DOI:** 10.31744/einstein_journal/2021AO5969

**Published:** 2021-07-22

**Authors:** Vitor Ulisses Monnaka, Carlos Augusto Cardim de Oliveira

**Affiliations:** 1 Faculdade Israelita de Ciências da Saúde Albert Einstein Hospital Israelita Albert Einstein São PauloSP Brazil Faculdade Israelita de Ciências da Saúde Albert Einstein, Hospital Israelita Albert Einstein, São Paulo, SP, Brazil.

**Keywords:** Communicable diseases, Epidemiological monitoring, Population surveillance, Search engine, Information technology, Forecasting, Yellow fever, Dengue

## Abstract

**Objective:**

To assess Google Trends accuracy for epidemiological surveillance of dengue and yellow fever, and to compare the incidence of these diseases with the popularity of its terms in the state of São Paulo.

**Methods:**

Retrospective cohort. Google Trends survey results were compared to the actual incidence of diseases, obtained from *Centro de Vigilância Epidemiológica “Prof. Alexandre Vranjac”*, in São Paulo, Brazil, in periods between 2017 and 2019. The correlation was calculated by Pearson’s coefficient and cross-correlation function. The accuracy was analyzed by sensitivity and specificity values.

**Results:**

There was a statistically significant correlation between the variables studied for both diseases, Pearson coefficient of 0.91 for dengue and 0.86 for yellow fever. Correlation with up to 4 weeks of anticipation for time series was identified. Sensitivity was 87% and 90%, and specificity 69% and 78% for dengue and yellow fever, respectively.

**Conclusion:**

The incidence of dengue and yellow fever in the State of São Paulo showed a strong correlation with the popularity of its terms measured by Google Trends in weekly periods. Google Trends tool provided early warning, with high sensitivity, for the detection of outbreaks of these diseases.

## INTRODUCTION

Communicable diseases are a threat to the health of individuals, especially in developing countries.^(1)^ In Brazil, dengue and yellow fever represent infections of great impact on the health of the population.^(2)^ Early identification of communicable disease outbreaks increases the possibility of spread control with the eventual use of prevention, isolation, and treatment interventions.^(3)^

Dengue fever is an acute infection, with high mortality rates,^(4)^ mainly transmitted by the mosquito *Aedes aegypti*,^(5)^ which has its cases classified as without warning signs, with warning signs and severe, according to the World Health Organization (WHO). It is the major cause of arbovirus in the world,^(6)^ and Brazil ranks first in number of cases, in this century.^(7,8)^ Yellow fever, an acute, febrile, hemorrhagic and non-contagious infection, and has accounted for high mortality in South American and African populations since the 17^th^ century.^(9,10)^ It presents two distinct patterns of epidemiological transmission: wild and urban – both by *Aedes aegypti.*^(10,11)^ According to the Epidemiological Bulletin volume 51, published by the Brazilian Ministry of Health, a total of 714,164 probable dengue cases were identified and 298 deaths due to dengue were confirmed from January to May 2020. Between July 2019 and May 2020, 812 cases of yellow fever were reported in the country, 324 of them in the State of São Paulo.

Due to the impact on the health of the Brazilian population, effective surveillance of dengue and yellow fever cases is extremely important for epidemic control.^(3)^ In the state of São Paulo, the *Centro de Vigilância Epidemiológica* (CVE) *“Prof. Alexandre Vranjac”*, a epidemiological surveillance agency in the structure of the Disease Control Coordination (CDC - *Coordenadoria de Controle de Doenças*), is responsible for the disclosure of periodic reports on the status of these diseases, by epidemiological weeks. However, the presence of an effective surveillance structure is not homogeneous for all Brazilian states, and underreporting is a possible failure factor for the accuracy of cases.

Considering the obstacles present in epidemiological surveillance of communicable diseases, online tools have been suggested as complementary methods to obtain information, which signals potential outbreaks. A study^(12)^ conducted with Brazilian data, using the Twitter tool, showed an association between tweets and dengue, demonstrating the possibility of this tool to estimate the number of cases weekly. The National Contingency Plan for Dengue Epidemics, prepared by the Ministry of Health, also guides the use of the relative trend of rumors on Twitter, as indicators for specific actions in response. In this sense, Google Trends,^(13)^ a tool that analyzes the popularity of a term searched on Google,^(14)^ over a period of time, in a location, could be useful in surveillance of dengue and yellow fever cases.

In Google Trends, the trend for a given term is displayed on a scale of zero to one hundred, in which on hundred represents the largest search volume for the term, at a given location and period. The results represent a relative value that reflects the number of searches performed for a specific term, compared to the total number of searches performed. In recent years, the attempt to use this instrument for health-related issues has been increasing. Previous studies have analyzed Google Trends’ ability to predict influenza epidemics in Latin America,^(15)^ and confirmed cases of Zika,^(16)^ and demonstrated correlation between the trends of the terms and the cases of dengue around the world.^(17-20)^

Thus, given the published evidence, it is important to evaluate the usefulness of this platform for epidemiological surveillance of dengue and yellow fever.

## OBJECTIVE

To assess Google Trends accuracy for epidemiological surveillance of dengue and yellow fever, and to compare the incidence of these diseases with the popularity of its terms in the state of São Paulo.

## METHODS

The project was carried out at *Faculdade Israelita de Ciências da Saúde Albert Einstein* (FICSAE - HIAE), from August 2018 to August 2019. This project did not require approval by the Research Ethics Committee, since it only used data in the public domain, not involving human beings. The design used was a retrospective cohort. Google Trends data were obtained from its online platform https://trends.google.com/trends/, which provides the trends related to search frequency of their terms on Google, on a scale of zero to one hundred, in which one hundred represents the largest search volume for the term, at a given location and time period. Results denoted as “<1” in terms of trends were approximated to the value of one, with the objective of quantitatively standardizing information, enabling statistical analysis. Information on yellow fever was obtained through the term “*febre amarela*” in the state of São Paulo, by epidemiological week, from January 1, 2017 to May 19, 2018, comprising a total of 70 weeks, 50 from 2017, plus 20 from 2018. Epidemiological weeks 24 and 26 weeks, from 2017, were excluded from the analysis due to the lack of publication of epidemiological bulletins for these periods. Information on dengue was obtained through the term “*dengue*” in the state of São Paulo, by epidemiological week, from December 31, 2017 to March 30, 2019, comprising a total of 65 weeks, 52 from 2018, plus 13 from 2019. The incidence of yellow fever and dengue in the state of São Paulo was obtained from the epidemiological bulletin released by the CVE of State Health Authority of São Paulo, representing the total number of cases in the corresponding periods.

### Statistical analysis

The association between the quantitative measurements of the methods was assessed using Pearson’s correlation coefficient and time series analysis.^(21)^ The cross-correlation function enables the assessment of temporal dependence between two series of variables through lag values, which express the degree and direction of the association. A lag of -2 for a given coefficient indicates that Google Trends data shifted back two weeks from CVE records. That is, the correlation of the increase of the trends is represented two weeks before the registration of the cases. Statistical analyses were performed using the software RStudio,^(22)^ and the significance level considered was 0.05.

The diagnostic accuracy of this tool for detecting epidemics in the state of São Paulo was assessed by classifying epidemiological weeks, by the presence or absence of epidemics, and trends in terms of Google Trends, by the presence or absence of warning signs. Based on CVE data, considered gold standard, we established as epidemic thresholds the case numbers of, at least, three and 500, for yellow fever and dengue, respectively. We considered as a warning signal the trends values of, at least, four and five, for yellow fever and dengue, respectively. The comparison between these data enabled calculation of sensitivity and specificity values.

## RESULTS

**Figure 1:**
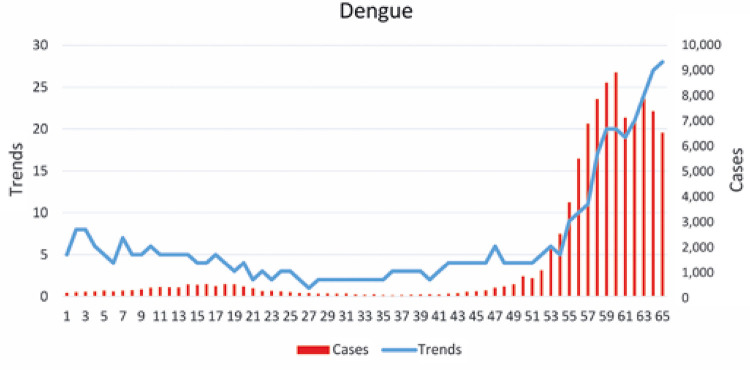
Cases and trends of dengue per epidemiological week

**Figure 2:**
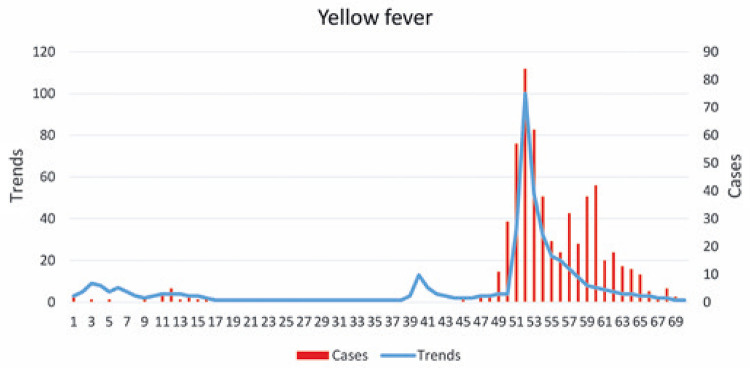
Cases and trends of yellow fever per epidemiological week

Figures 1 and 2 represent the incidence of diseases and their Google Trends terms for dengue and yellow fever, respectively. In figure 1, the dengue epidemic was determined as from week 50, when there were 798 cases. In figure 2, there are two epidemics of yellow fever, one starting at week 11, and another, at week 50, both with three cases.

**Figure 3:**
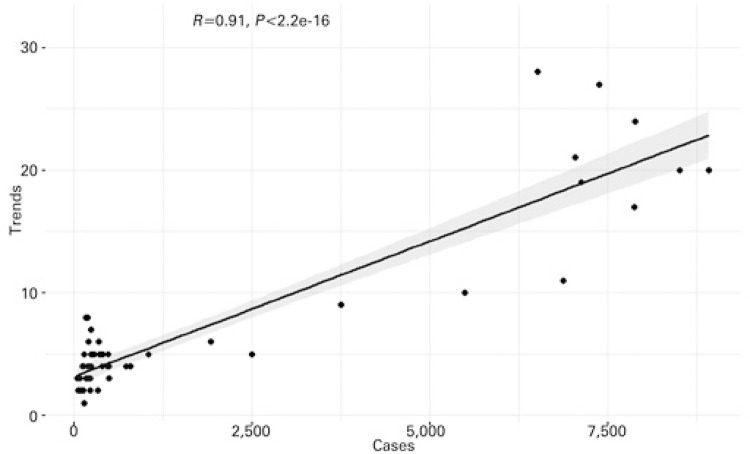
Scatter plot of dengue. Statistics represent Pearson’s correlation test

**Figure 4:**
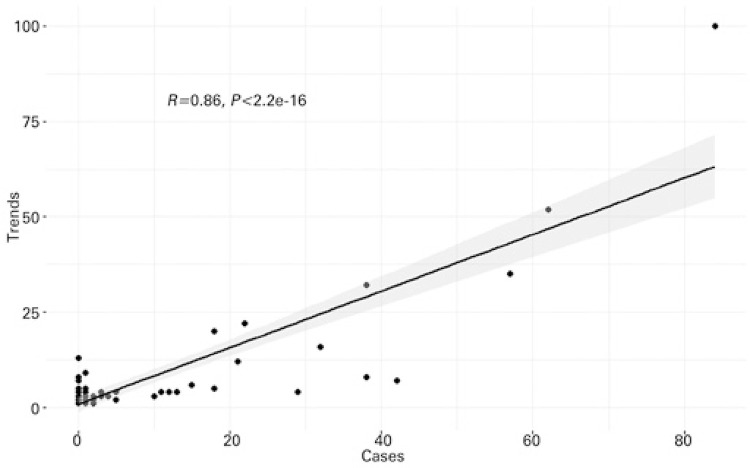
Scatter plot of yellow fever. Statistics represent Pearson’s correlation test

Figures 3 and 4 represent scatter plots between incidence of disease and Google Trends search terms for dengue and yellow fever, respectively. There was a statistically significant correlation (p<0.0001) for both diseases. In the case of dengue, Pearson’s coefficient was 0.91, while for yellow fever, the coefficient was 0.86. The results of these analyzes are shown in table 1. Cross-correlation analysis showed a statistically significant correlation for up to 4 weeks of displacement between time series, as shown in table 2.

**Table 1:** Results of Pearson correlation tests

Diseases	Coefficient	95%CI
Dengue	0.91	0.86-0.94
Yellow fever	0.86	0.78-0.91

95%CI: confidence interval 95%. Correlation between the incidence of these two diseases and the search results in Google Trends for terms related to them.

**Table 2:** Cross-correlation coefficients

Lag in weeks	Dengue	Yellow fever
-4	0.46*	0.27*
-3	0.56*	0.30*
-2	0.67*	0.45*
-1	0.79*	0.68*
0	0.91*	0.86*

* Significant in p≤0.05. Correlation between the incidence of these two diseases and the search results in Google Trends for terms related to them with the assessment of temporal dependence through lag values.

To assess the accuracy of Google Trends for epidemic detection, the values of the true positive, false positive, true negative and false negative were calculated, as shown in table 3. From these data, a sensitivity of 87% and a specificity of 69% were calculated for dengue, and a sensitivity of 90% and a specificity of 78%, for yellow fever.

**Table 3:** Crossover for diagnostic accuracy evaluation

Dengue			Yellow fever		
Google Trends alert	Epidemic		Google Trends alert	Epidemic	
	Present	Absent		Present	Absent
Present	14	15	Present	18	10
Absent	2	34	Absent	2	35

Comparison between the classification of epidemiological weeks by the presence or absence of epidemics and trends in terms of Google Trends by the presence or absence of warning signs.

## DISCUSSION

Google Trends allows evaluating human behavior and predicting health-related issues, and it has been demonstrated the seasonality found in online searches is related to cases of the surveyed diseases.^(23)^ Statistical methods and approaches for this type of analysis have already been described in systematic reviews.^(24)^

The present study demonstrated the data obtained with this tool showed a strong correlation with the incidence of yellow fever in the state of São Paulo, in the evaluation in weekly periods. The high correlation between dengue cases and Google Trends has already been identified in Indonesia,^(18)^ Philippines,^(19)^ and India.^(20)^ Using Brazilian data, Yang et al.,^(17)^ compared surveys with dengue cases provided by the Ministry of Health, on monthly periods, between January 2001 and December 2012, and found a correlation of 0.971, similar to our results. However, this study is the first to demonstrate this correlation with Brazilian data on a weekly basis. This approach enabled evaluating time series already performed in other countries,^(18-20)^ and showed a moderate correlation before epidemics occurred, with up to 4 weeks difference for dengue, and 3 weeks for yellow fever. This indicates the ability of this tool to provide an early warning, enabling authorities to take action to anticipate the spread of these diseases.

Other studies have evaluated Google Trends using Brazilian data from other diseases. One study^(15)^ assessed the predictive capacity of influenza epidemics in Latin America, comparing the proportion of cases on the FluNet platform, between January 2011 and December 2014, with Google Trends data, obtaining Pearson´s correlation coefficients between 0.48 in 2012 and 0.61 in 2014, in Brazil. This article found a substantial inaccuracy of Google Trends compared to FluNet, most likely due to limited Internet access in some regions. It also highlighted the limitations of FluNet due to the geographic dimensions of Brasil, as well as to its ecological and demographic diversity. Another study^(16)^ analyzed the predictive capacity of confirmed cases of Zika in Brazil, showing that Google Trends could anticipate the epidemic a week in advance.

Unlike the study by Marques-Toledo et al.,^(12)^ who developed a model for predicting the number of dengue cases based on data from the Twitter social network, this study proposes a different use of online tools. We believe that the greatest importance of these instruments is in identifying the occurrence of an epidemic, and not necessarily in predicting the number of cases. Hence, this study is the first to analyze the accuracy of Google Trends to identify outbreaks. A high sensitivity was found for yellow fever (0.90) and dengue (0.87), which points to a practical utility of this tool, especially noting that, in the case of prediction, sensitivity is more useful than specificity, for it indicates a low probability of false negatives, less likely to lose cases. Thus, its applicability can be very useful, especially in states with less effective epidemiological surveillance systems, as a complementary analysis to the available methods.

Another useful health-related utility of Google Trends could be the assessment of diseases that are not within the scope of epidemiological surveillance agencies. Its usefulness could also be significant for monitoring uncommon adverse reactions, and new beneficial effects for medications, as well as evaluating effective dose minimization (Phase IV studies), after drug marketing.

### Limitations

Some limitations may be noted for the methods of analysis employed in this study, to verify the incidence of the disease as a cause of the increase in trends of related terms, due to the existence of other mechanisms of association between two variables, such as chance or confusion. As trends of the Google Trends are determined by the interests of Internet users, they may produce a random correlation, not necessarily due to the incidence of diseases. However, the large volume of research significantly reduces the probability of error due to chance. In addition, there may be other variables responsible for inducing positive confusion, such as awareness campaigns conducted at the time of the highest incidence of the disease or media news, which increases public interest and, consequently, the search rate.

Since the use of Google’s search tool depends on Internet access, we also emphasize that less favored regions may have lower search rates, even with a high incidence of a certain disease, which limits the applicability of this instrument.

## CONCLUSION

The study showed a significant correlation between the data generated by the Google Trends tool and the incidence of dengue and yellow fever in the state of São Paulo in the weekly period evaluation. The increased search provided early warning for outbreaks of these diseases, and showed high sensitivity for detecting epidemics. Further research should be conducted to confirm these findings for other diseases and locations, but the findings suggest the possibility of employing this tool as a simple and inexpensive method for epidemiological surveillance.
