# Supplementary material for: Google Trends correlation and sensitivity for outbreaks of dengue and yellow fever in the state of São Paulo
Source: Einstein (Sao Paulo). 2021 Jul 22;19:eAO5969. doi: 10.31744/einstein_journal/2021AO5969 (PMC8302225; doi:10.31744/einstein_journal/2021AO5969)
Supplement: Translation

# Translation: Correlação e sensibilidade do *Google Trends* para surtos de dengue e febre amarela no estado de São Paulo

**Authors:** Vitor Ulisses Monnaka, Carlos Augusto Cardim de Oliveira

**Affiliations:** 1 Brasil Faculdade Israelita de Ciências da Saúde Albert Einstein, Hospital Israelita Albert Einstein, São Paulo, SP, Brasil.

**Keywords:** Doenças transmissíveis, Monitoramento epidemiológico, Vigilância da população, Ferramenta de busca, Tecnologia da informação, Previsões, Febre amarela, Dengue

## Abstract

**Objetivo:**

Avaliar a acurácia do *Google Trends* para vigilância epidemiológica de dengue e febre amarela e comparar a incidência dessas doenças com a popularidade de seus termos no estado de São Paulo.

**Métodos:**

Coorte retrospectiva. Os resultados da pesquisa *Google Trends* foram comparados com a incidência real de doenças, obtida do Centro de Vigilância Epidemiológica “Prof. Alexandre Vranjac”, do estado de São Paulo, nos períodos entre 2017 e 2019. A correlação foi calculada pelo coeficiente de Pearson e pela função de correlação cruzada. A acurácia foi analisada por valores de sensibilidade e especificidade.

**Resultados:**

Houve correlação estatisticamente significante entre as variáveis estudadas para ambas as doenças, com coeficiente de Pearson de 0,91 para dengue e 0,86 para febre amarela. Foi identificada correlação com até 4 semanas de antecipação para séries temporais. A sensibilidade foi de 87% e 90% e a especificidade de 69% e 78% para dengue e febre amarela, respectivamente.

**Conclusão:**

A incidência de dengue e febre amarela no estado de São Paulo apresentou forte correlação com a popularidade de seus termos medidos pelo *Google Trends* em períodos semanais. A ferramenta *Google Trends* forneceu alerta precoce, com alta sensibilidade, para a detecção de surtos dessas doenças.

## INTRODUÇÃO

As doenças transmissíveis são uma ameaça à saúde dos indivíduos, principalmente nos países em desenvolvimento.^(1)^ No Brasil, a dengue e a febre amarela são infecções de grande impacto na saúde da população.^(2)^ A identificação precoce de surtos de doenças transmissíveis aumenta a possibilidade de controle da disseminação, com uso provável em intervenções para prevenção, isolamento e tratamento.^(3)^

A dengue é uma infecção aguda, com altas taxas de mortalidade,^(4)^ transmitida predominantemente pelo mosquito *Aedes aegypti*,^(5)^ cujos casos são classificados como sem sinais de alerta, com sinais de alerta e graves, de acordo com a Organização Mundial da Saúde (OMS). É a principal infecção por arbovírus no mundo,^(6)^ e o Brasil é o país com o maior número de casos nesse século.^(7,8)^ A febre amarela, uma infecção aguda, febril, hemorrágica e não contagiosa, responde por alta mortalidade na população da América do Sul e da África, desde o século 17.^(9,10)^ Ela se manifesta em dois padrões de transmissão epidemiológica distintos: selvagem e urbano – ambos pelo *Aedes aegypti.*^(10,11)^ De acordo com o Boletim Epidemiológico volume 51, publicado pelo Ministério da Saúde do Brasil, 714.164 casos prováveis de dengue foram identificados, e 298 mortes por dengue foram confirmadas de janeiro a maio de 2020. Entre julho de 2019 e maio de 2020, 812 casos de febre amarela foram registrados no país, dos quais 324 ocorreram no estado de São Paulo.

Devido ao impacto na saúde da população brasileira, a vigilância efetiva de casos de dengue e febre amarela é extremamente importante para o controle de epidemias.^(3)^ No estado de São Paulo, o Centro de Vigilância Epidemiológica (CVE) “Prof. Alexandre Vranjac”, instituição subordinada à estrutura da Coordenadoria de Controle de Doenças (CDC), é responsável pela divulgação de relatórios periódicos sobre o *status* dessas doenças, por semana epidemiológica. No entanto, a presença de uma estrutura de vigilância efetiva não é homogênea em todos os estados do Brasil, e a subnotificação é um possível fator de falha para acurácia do número de casos.

Considerando-se os obstáculos presentes na vigilância epidemiológica de doenças transmissíveis, já foi sugerido o uso de ferramentas *on-line* como métodos complementares para obtenção de informações, que sinalizem possíveis surtos de doenças. Um estudo^(12)^ com dados brasileiros usando a ferramenta *Twitter* demonstrou associação entre *tweets* e a dengue e levantou a possibilidade de essa ferramenta ser usada para estimar o número de casos semanalmente. O Plano de Contingência Nacional para Epidemias de Dengue, elaborado pelo Ministério da Saúde, também menciona o uso de tendências relativas de boatos no *Twitter* como indicadores para ações de resposta específicas. Nesse sentido, o *Google Trends*,^(13)^ ferramenta que analisa a popularidade de termos de busca no *Google*,^(14)^ em períodos e locais determinados, poderia ser útil para a vigilância de casos de dengue e febre amarela.

No *Google Trends*, a tendência de um determinado termo é exibida em uma escala de zero a cem, em que cem representa o maior volume de buscas para o termo em questão, em determinado local e período. Os resultados representam um valor relativo, que reflete o número de buscas realizadas para um termo específico, comparado ao total de número de buscas realizadas. Recentemente, as tentativas de usar esse instrumento para questões relativas à saúde têm sido mais frequentes. Estudos anteriores já analisaram a capacidade do *Google Trends* de prever epidemias de gripe na América Latina^(15)^ e casos confirmados de Zika^(16)^ e demonstraram correlação entre as tendências de termos e os casos de dengue no mundo.^(17-20)^

Sendo assim, considerando-se as evidências já publicadas, é importante avaliar a utilidade dessa plataforma para vigilância epidemiológica da dengue e da febre amarela.

## OBJETIVO

Avaliar a acurácia do *Google Trends* para vigilância epidemiológica da dengue e da febre amarela e comparar a incidência dessas doenças com a popularidade de seus termos no estado de São Paulo.

## MÉTODOS

O projeto foi realizado na Faculdade Israelita de Ciências da Saúde Albert Einstein (FICSAE - HIAE), de agosto de 2018 a agosto de 2019. Este projeto não precisou de aprovação do Comitê de Ética em pesquisa, porque usou somente dados de domínio público, sem envolver seres humanos. O desenho usado foi o de coorte retrospectiva. Os dados do *Google Trends* foram obtidos na plataforma *on-line* https://trends.google.com/trends/, que informa as tendências relacionadas à frequência de buscas dos respectivos termos no *Google*, em uma escala de zero a cem, na qual cem representa o maior volume de buscas pelo termo, em um determinado local e período. Os resultados representados como “<1” em termos de tendência foram arredondados para o valor de umx, com o objetivo de padronizar quantitativamente as informações, permitindo a análise estatística. Informações sobre a febre amarela foram obtidas com base no termo “febre amarela” no estado de São Paulo, por semana epidemiológica, de 1° de janeiro de 2017 a 19 de maio de 2018, em um total de 70 semanas, sendo 50 de 2017 e 20 de 2018. As semanas epidemiológicas 24 e 26, de 2017, foram excluídas da análise devido à falta de publicação de boletins epidemiológicos para esses períodos. Informações sobre a dengue foram obtidas com base no termo “dengue” no estado de São Paulo, por semana epidemiológica, de 31 de dezembro de 2017 a 30 de março de 2019, em um total de 65 semanas, sendo 52 de 2018 e 13 de 2019. A incidência de febre amarela e dengue no estado de São Paulo foi obtida no boletim epidemiológico divulgado pelo CVE da Secretária de Saúde do Estado de São Paulo, que lista o número total de casos nos períodos correspondentes.

### Análise estatística

A associação entre as medições quantitativas dos métodos foi avaliada usando-se o coeficiente de correlação de Pearson e a análise de séries históricas.^(21)^ A função de correlação cruzada permite avaliar a dependência temporal entre as séries de variáveis por meio dos valores de *lag*, que expressam o grau e a direção da associação. Um *lag* de -2 para um determinado coeficiente indica que os dados do *Google Trends* corresponderam a 2 semanas a menos que os registros do CVE. Isso quer dizer que a correlação do aumento nas tendências é notada 2 semanas antes do registro dos casos. Foram realizadas análises estatísticas com o programa RStudio,^(22)^ e o nível de significância considerado foi 0,05.

A acurácia diagnóstica da ferramenta para detecção de epidemias no estado de São Paulo foi avaliada pela classificação das semanas epidemiológicas, quanto à presença ou à ausência de epidemias, e tendências nos termos do *Google Trends*, pela presença ou ausência de sinais de alerta. Com base nos dados de CVE, considerados o padrão-ouro, estabelecemos como limiares epidêmicos os números de casos de, no mínimo, três e 500, para febre amarela e dengue, respectivamente. Consideramos como sinal de alerta valores de tendências de, no mínimo, quatro e cinco, para febre amarela e dengue, respectivamente. A comparação entre esses dados permitiu o cálculo dos valores de sensibilidade e especificidade.

## RESULTADOS

As figuras 1 e 2 representam a incidência de doenças e seus termos no *Google Trends* para dengue e febre amarela, respectivamente. Na figura 1, a epidemia de dengue foi determinada a partir da semana 50, quando ocorreram 798 casos. Na figura 2, há duas epidemias de febre amarela, uma começando na semana 11 e outra na semana 50, ambas com três casos.

**Figura 1:** Casos e *trends* para dengue, por semana epidemiológica

**Figura 2:** Casos e *trends* para febre amarela, por semana epidemiológica

As figuras 3 e 4 representam gráficos de dispersão entre a incidência da doença e os termos de busca no *Google Trends* para dengue e febre amarela, respectivamente. Houve correlação estatisticamente significativa (p<0,0001) para ambas as doenças. No caso da dengue, o coeficiente de Pearson foi 0,91; para a febre amarela, o coeficiente foi 0,86. Os resultados dessas análises são mostrados na tabela 1. A análise de correlação cruzada mostrou correlação estatisticamente significativa para até 4 semanas de deslocamento entre as séries históricas, conforme mostrado na tabela 2.

**Figura 3:** Gráfico de dispersão para dengue. As estatísticas representam o teste de correlação de Pearson

**Figura 4:** Gráfico de dispersão para febre amarela. As estatísticas representam o teste de correlação de Pearson

**Tabela 1:** Resultados dos testes de correlação de Pearson

Doença	Coeficiente	IC95%
Dengue	0,91	0,86-0,94
Febre amarela	0,86	0,78-0,91

IC95%: intervalo de confiança de 95%. Correlação entre a incidência dessas doenças e os resultados de buscas no *Google Trends* para termos a elas relacionados.

**Tabela 2:** Coeficientes de correlação cruzada

*Lag* em semanas	Dengue	Febre amarela
-4	0,46*	0,27*
-3	0,56*	0,30*
-2	0,67*	0,45*
-1	0,79*	0,68*
0	0,91*	0,86*

* Significativo em p≤0,05. Correlação entre a incidência dessas doenças e os resultados de buscas no *Google Trends* para termos a elas relacionados, com avaliação da dependência temporal com base em valores de *lag*.

Para avaliar a acurácia do *Google Trends* para detecção de epidemias, os valores de positivos-verdadeiros, falsos-positivos, negativos-verdadeiros e falsos-negativos foram calculados, conforme mostrado na tabela 3. A partir desses dados, foram calculadas sensibilidade de 87% e especificidade de 69% para dengue, e sensibilidade de 90% e especificidade de 78%, para febre amarela.

**Tabela 3:** Cruzamento para avaliação de acurácia diagnóstica

Dengue			Febre amarela		
Alerta do *Google Trends*	Epidemia		Alerta do *Google Trends*	Epidemia	
	Presente	Ausente		Presente	Ausente
Presente	14	15	Presente	18	10
Ausente	2	34	Ausente	2	35

Comparação entre a classificação de semanas epidemiológicas, pela presença ou ausência de epidemias, e tendências nos termos do *Google Trends*, pela presença ou ausência de sinais de alerta.

## DISCUSSÃO

O *Google Trends* permite a avaliação do comportamento humano e a previsão de problemas de saúde, tendo sido foi demonstrado que a sazonalidade observada nas buscas *on-line* está relacionada a casos das doenças estudadas.^(23)^ Métodos estatísticos e abordagens para esse tipo de análise já foram descritos em revisões sistemáticas.^(24)^

O presente estudo demonstrou que os dados obtidos com a ferramenta tiveram forte correlação com a incidência de febre amarela no estado de São Paulo, na avaliação em intervalos semanais. Essa alta correlação entre os casos de dengue e o *Google Trends* já foi identificada na Indonésia,^(18)^ nas Filipinas^(19)^ e na Índia.^(20)^ Usando dados do Brasil, Yang et al.,^(17)^ compararam levantamentos de casos de dengue fornecidos pelo Ministério da Saúde, mensalmente, de janeiro de 2001 a dezembro de 2012, e encontraram correlação de 0,971, semelhante ao resultado da presente pesquisa. No entanto, este estudo é o primeiro a demonstrar essa correlação com dados brasileiros de base semanal. Essa abordagem possibilitou avaliação de séries históricas já realizadas em outros países^(18-20)^ e demonstrou correlação moderada antes que a epidemia ocorresse, com até 4 semanas de diferença para dengue e 3 semanas para febre amarela. Isso indica que a ferramenta tem a habilidade de fornecer um alerta precoce, permitindo que as autoridades tomem medidas para prever a propagação dessas doenças.

Outros estudos avaliaram o *Google Trends* usando dados brasileiros de outras doenças. Um estudo^(15)^ avaliou a capacidade de previsão de epidemias de gripe na América Latina, comparando-se a proporção de casos na plataforma *FluNet*, entre janeiro de 2011 e dezembro de 2014, com dados do *Google Trends*, e os coeficientes de correlação de Pearson estiveram entre 0,48, em 2012, e 0,61, em 2014, no Brasil. Este artigo encontrou imprecisão considerável do *Google Trends* comparada a *FluNet*, muito provavelmente devido ao acesso limitado à Internet em algumas regiões. Destacou também as limitações da *FluNet* devido à dimensão geográfica do Brasil, bem como por sua diversidade ecológica e demográfica. Outro estudo^(16)^ analisou a capacidade de previsão de casos confirmados de Zika no Brasil e demonstrou que o *Google Trends* conseguiu prever a epidemia com 1 semana de antecipação.

Diferente do estudo de Marques-Toledo et al.,^(12)^ que desenvolveram um modelo para prever o número de casos de dengue com base em dados da rede social *Twitter*, este estudo propõe um uso diferente das ferramentas *on-line*. Acredita-se que a maior importância desses instrumentos está na identificação da ocorrência de epidemias, e não necessariamente na previsão do número de casos. Portanto, este estudo é o primeiro a analisar a acurácia do *Google Trends* na identificação de surtos de doenças. Foi observada alta sensibilidade para febre amarela (0,90) e dengue (0,87), indicando utilidade prática da ferramenta, principalmente porque, para fins de previsão, a sensibilidade é mais útil que a especificidade, já que a primeira indica baixa probabilidade de falsos-negativos e de casos perdidos. Portanto, a ferramenta pode ser muito útil, principalmente em estados com sistemas de vigilância epidemiológica menos eficazes, como uma análise complementar aos métodos disponíveis.

Outra utilidade relevante do *Google Trends* relativa à saúde poderia ser a avaliação de doenças que não estejam no escopo das instituições de vigilância epidemiológica. A ferramenta também poderia ser consideravelmente útil para o monitoramento de reações adversas incomuns e novos efeitos benéficos de medicamentos, bem como para avaliar a minimização de doses efetivas (estudos de Fase IV), após a inserção de medicamentos no mercado.

### Limitações

Existem algumas limitações nos métodos de análise empregados neste estudo, para verificar a incidência da doença como causa do aumento das tendências de termos relacionados, devido à existência de outros mecanismos de associação entre duas variáveis, como acaso ou confusão. Como as tendências do *Google Trends* são determinadas pelos interesses dos usuários da Internet, elas podem produzir uma correlação aleatória, não necessariamente decorrente da incidência das doenças. No entanto, o maior volume de pesquisas reduz significativamente a probabilidade de erros, devido ao fator acaso. Além disso, pode haver outras variáveis responsáveis por induzir uma confusão positiva, como campanhas de conscientização conduzidas no momento de pico da incidência da doença ou menções na mídia, o que aumenta o interesse público e, consequentemente, a frequência de buscas.

Enfatiza-se também que, como o uso da ferramenta *Google Trends* depende do acesso à Internet, regiões menos favorecidas apresentam taxas de busca mais baixas, mesmo com alta incidência de determinada doença, o que limita a aplicabilidade deste instrumento.

## CONCLUSÃO

O estudo demonstrou correlação significativa entre os dados gerados pela ferramenta *Google Trends* e a incidência de dengue e febre amarela no estado de São Paulo na avaliação em intervalos semanais. A maior frequência de buscas serviu como alerta precoce para surtos dessas doenças e demonstrou alta sensibilidade para a detecção de epidemias. São necessárias mais pesquisas para confirmar estes achados para outras doenças e localidades, mas as conclusões apontam para a possibilidade de se usar essa ferramenta como método simples e barato de vigilância epidemiológica.
